# Dereplication of Cytochalasans and Octaketides in Cytotoxic Extracts of Endophytic Fungi from *Casearia arborea* (Salicaceae)

**DOI:** 10.3390/metabo12100903

**Published:** 2022-09-26

**Authors:** Augusto L. Santos, Marisa Ionta, Renato O. Horvath, Marisi G. Soares, Daniele O. Silva, Eunizinis S. Kawafune, Marcelo J. P. Ferreira, Patricia Sartorelli

**Affiliations:** 1 Institute of Environmental, Chemical and Pharmaceutical Sciences, Federal University of São Paulo, São Paulo 09972-270, Brazil; 2 Institute of Biomedical Science, Federal University of Alfenas, Minas Gerais 37130-000, Brazil; 3 Institute of Chemistry, Federal University of Alfenas, Minas Gerais 37130-000, Brazil; 4 Botany Department, Institute of Biosciences, University of São Paulo, São Paulo 05508-090, Brazil

**Keywords:** cytosporone, dothiorelone, cytochalasin, *Phomopsis*, *Diaporthe*, GNPS

## Abstract

Endophytes have been shown to be a source of novel drug prototypes. The *Casearia* genus is known for presenting cytotoxic clerodane diterpenes; however, there are few reports on secondary metabolites produced by its fungal microbiota. Thus, in the present study endophytic fungi obtained from the fresh leaves of *C. arborea* were grown in potato dextrose broth and rice to perform a secondary metabolite prospection study. The cytotoxic profile of the crude extracts at 10 µg/mL was determined by a colorimetric assay on tumor cell lines. The endophytes producing cytotoxic extracts were identified through phylogenetic analysis and belong to *Diaporthe* and *Colletotrichum* species. Metabolites present in these extracts were organized in molecular networking format based on HRMS-MS, and a dereplication process was performed to target compounds for chromatographic purification. Metabolic classes, such as lipids, peptides, alkaloids, and polyketides were annotated, and octaketide and cytochalasin derivatives were investigated. Cytochalasin H was purified from the cytotoxic *Diaporthe* sp. CarGL8 extract and its cytotoxic activity was determined on human cancer cell lines A549, MCF-7, and HepG2. The data collected in the present study showed that molecular networking is useful to understand the chemical profile of complex matrices to target compounds, minimizing the cost and time spent in purification processes.

## 1. Introduction

The *Casearia* genus (Salicaceae) comprises species with pharmacological properties, which have been used in traditional medicine. *C. sylvestris* displays anti-inflammatory, anti-ulcer, anti-ophidian, and antitumor activities [1,2]. Highly oxidized clerodane diterpenes are very known and investigated in *Casearia* due to their high cytotoxic effects against several tumor cell lines representing promising anticancer natural prototypes [3,4,5].

Secondary metabolites from endophytes, especially those produced by fungi, may be useful as anticancer agents [6,7,8]. The endophytic microorganisms establish interactions with plant hosts and live inside their internal tissues, apparently causing no harm to the host as well as contributing to plant development [9,10,11] *Diaporthe* spp. is currently associated as a plant pathogen that has been extensively investigated due to its ability to produce several secondary metabolites with biological activities including antibacterial, anticancer, antifungal, antimalarial, antiviral, and herbicidal [12]. The *Colletotrichum* genus is responsible for causing plant diseases, such as anthracnose, in crops worldwide [13,14,15,16]; however, it may be found as non-pathogen endophytes [17]. Besides the pathogenicity and phytotoxic secondary metabolites, *Colletotrichum* has been placed among the top 10 fungi genera with economic and scientific importance [18].

Until now, a few endophytic fungi from *Casearia* species were investigated regarding their metabolites. These studies include alkaloids and polyketides derivatives from *Colletotrichum crassipes*, as well as diketopiperazines and simple phenolics from *Xylaria* sp., both fungi isolated from *C. sylvestris* [19], and a diterpenoid isolated from *Neosartorya fischeri*, an endophyte from *C. grewiifolia* [20]. Additionally, *Phomopsis* sp. from *C. arborea* produced octaketides, known as cytosporones and dothiorelones [21]. The *Casearia* mycobiota is still unknown and represents a source to identify fungal metabolites of biological interests. Herein, we report the cytotoxic potential of *Casearia arborea* endophytes in a colorimetric assay to guide liquid chromatography and mass spectrometry analysis. The online platform Global Natural Products Social Molecular Networking (GNPS) was chosen as a modern tool for spectral organization of metabolites detected in cytotoxic extracts obtained from the incubation of *Colletotrichum* sp., *Diaporthe* spp., and *Phomopsis* sp. to perform the molecular networking (MN). Modern tools, such as MN facilitate annotation processes to aid the chemical profile identification of complex matrixes. Bio-guided assays and MN help to target compounds for chromatographic purification, as described in this work for cytochalasin H from a cytotoxic *Diaporthe* sp. extract incubation.

## 2. Materials and Methods

### 2.1. Plant Material

Leaves of *Casearia arborea* Rich. were collected in the Atlantic Forest, Alfenas city, MG, Brazil (coordinates S 21°22′53.8″ W 045°55′46.4″), in June 2016. The botanical identification was performed by João Pedro Costa Elias from the Federal University of Alfenas (UNIFAL—Alfenas city, MG, Brazil), and a voucher specimen (Elias J.P.C. 02) was deposited in the SPF herbarium, São Paulo University.

### 2.2. Isolation of Endophytic Fungi

The methodology used for the isolation of endophytic fungi was based on the procedure described in the literature [22], as well as extraction procedures from liquid and solid cultures [23]. Briefly, healthy leaves were washed in running water and dipped successively into solutions of 70% ethanol (3 min), 1% hypochlorite (1 min), 70% ethanol. After the asepsis, the plant material was transferred to a laminar flow cabinet (Pachane, class Pa40) and dipped in ultra-pure water to remove hypochlorite and alcohol residues. The washed leaves were cut (1–3 mm^2^) and inoculation was performed in potato dextrose agar (PDA—Kasvi^®^) containing ampicillin (50 mgL^−1^) for fungi growth. The Petri dishes were sealed and the incubation was performed in an incubator at 25 °C. Each morphologically different mycelium that grew out from the plant fragment was subcultured to a plate with PDA medium for the isolation of strains codified as CarGL.

### 2.3. Identification of Endophytic Fungi

The mycelium grown on PDA was ground in liquid nitrogen. The genomic DNA was extracted using the “Wizard^®^ Genomic DNA Purification Kit” (Promega, Madison, WI, USA). The quality of the reaction was evaluated on agarose gel (1% *w*/*v*) with “SYBR^®^ Safe DNA Gel Stain” (Thermo Fisher Scientific, Waltham, MA, USA). The ITS1-5.8S-ITS2 region of ribosomal DNA was amplified with the universal primers for the Fungi kingdom, ITS-1 (5′TCCGTAGGTGAACCTGCGG-3′) and ITS-4 (5′TCCTCCGCTTATTGATATGC-3′), which amplify a region of approximately 600 bp. The reactions were prepared in a final volume of 50 μL containing genomic DNA, 1X enzyme buffer, 0.2 mM dNTPs, 0.2 μM primer ITS-1, 0.2 μM primer ITS-4 and 2.5 U/μL of Easy^®^ Taq DNA polymerase. The thermal cycler was programmed for an initial denaturation of 5 min at 94 °C, followed by 30 cycles of 94 °C for 30 s; 30 s at 55 °C; 1 min at 72 °C and a final extension of 10 min at 72 °C in a thermal cycler (Veriti 96-Well Thermal Cycler, 0.2 ML, Applied Biosystems, Waltham, MA, USA) [24]. Based on the ITS tree, the isolated fungi from *C. arborea* clustered together with fourteen species of *Diaporthe*, in addition to one *Colletotrichum* sp. The identification was performed using GenBank, Mycobank and BOLD databases (GenBank accession no. MT893333). A Diaporthaceae species (*Diaporthella corylina*) was used as an external group to construct a phylogenetic tree. The strains CarGL21, 39 and 42 were identified as *Diaporthe paranensis*. Twelve endophytes were assigned as *Diaporthe* sp. to strains CarGL2, 5, 8, 12, 18, 19, 30, 31, 35, 36, 37, 46, including strain *Phomopsis* sp. CarGL23 (Supplementary Materials SI), once *Phomopsis* is the asexual state of *Diaporthe* comprising hundreds of species belonging to Diaporthaceae [12].

### 2.4. Secondary Fungal Metabolite Prospection

Two culture media were used for endophytic fungi growth: potato dextrose broth (PDB—Kasvi^®^, liquid medium) and rice (“Uncle Ben’s”—parboiled, solid medium). For PDB incubation five fragments of each isolated fungus mycelium (3 mm^2^) from PDA incubation were inoculated in Erlenmeyer flasks (500 mL) containing 200 mL of PDB medium previously sterilized. Cultures were maintained on growth at 28 °C on a rotary shaker (120 rpm). The rice medium was prepared in Erlenmeyer flasks (500 mL) containing 50 g of rice and 90 mL of ultra-pure water followed by sterilization (121 °C, 20 min). After 5 days of incubation in PDB 5 mL was transferred to rice for solid incubation at 28 °C under static conditions. After 28 days both fermentations, in rice and PDB, were stopped by adding 200 mL of EtOAc to an Erlenmeyer flask and 20 min in an ultrasonic bath. Extraction was completed under starring on a shaker (120 rpm) for 24 h and then filtered. The extraction procedure was repeated two times. The filtrate was dried in a rotatory evaporator under low pressure at 40–50 °C furnishing the crude EtOAc extracts.

### 2.5. UPLC-ESI-HRMS-MS Data Acquisition

Liquid chromatography coupled to high-resolution tandem mass spectra (LC-HRMS-MS) data were acquired on Shimadzu Nexera X2 ultra-performance liquid chromatography system (Shimadzu, Japan) equipped with an SPD-M20A Proeminence Diode Array detector, using a reverse phase Kinetex EVO C18 column (2.6 µm—100.0 mm × 2.1 mm). All solvents were spectroscopic grade. The LC system was coupled to a QTOF mass spectrometer equipped with an electrospray (ESI) operating in positive ion mode at 18,000 FWHM of mass resolution (MicrOTOF-QII; Bruker Daltonics, Billerica, MA, USA).

Dried EtOAc crude extracts (5 mg) were dissolved in 1 mL of MeOH and centrifuged at 15,000 rpm for 10 min, 20 °C. The supernatant (500 µL) was transferred to a vial and the same volume of MeOH:H_2_O (1:1) was added. Compounds (1 mg mL^−1^) previously obtained from *Phomopsis* sp. CarGL23 [21] were used to guide the cytosporone and dothiorelone derivatives annotation in the molecular network. Samples were injected (3 µL) into the LC system at 50 °C in a column chamber. The chromatographic separation was performed using the mobile phase in a gradient of A (H_2_O + 0.1% formic acid) and B (ACN + 0.1% formic acid), and a flow of 350 µL min^−1^ for the following method: 0–2 min 5% B, 2–13 min 5% to 98% B, 13–16 min 98% B, 16–18 min 98% to 5% B, 18–21 min 5% B for the column stabilization for the next injection. HRMS-MS data were obtained from a quadrupole tandem time-of-flight (QTOF) mass analyzer under positive mode ESI at a mass range of *m*/*z* 50–1200. The positive ionization on ESI was set as follows: capillarity voltage of 4500 V and end plate offset at 500 V, dry gas (N_2_) at a flow of 8.0 mL min^−1^, a pressure of 4.0 Bar, and temperature of 200 °C. The collision-induced dissociation (CID) energy was set at 25 eV and auto-MS-MS were performed for three precursor ions, with active exclusion after three spectra and release after 1 min, reconsidering the precursor if the current intensity is five times more intense than the previous intensity. Calibration was set at less than 2 ppm using sodium formate.

### 2.6. HRMS-MS Data Organization

All data obtained from Bruker micrOTOF-QII were converted to the “.mzML” extension, to perform the dereplication on GNPS for database matches, using MSConvert [25] and conferred in SeeMS, both are free software from Proteowizard^®^. The “.mzML” archives were uploaded to the Global Natural Products Social Molecular Network Web server using WinSCP to create the molecular network [26]. The data were treated within the GNPS Data Analysis platform removing fragments of ±17 Da of precursor *m*/*z*. HRMS-MS spectra were filtered choosing only the six top fragments in the ±50 Da window in all ranges of spectra. In the basic options, the mass tolerance for precursor ions and fragment ions was set to 0.02 Da. A network was created using the MS-Cluster with a minimum cluster size containing two spectra [27] according to a cosine score above 0.7 and more than three matched peaks. Further, edges between two nodes were kept in the network if, and only if, each of the nodes appeared in each other’s respective top 10 most similar nodes. The maximum size of a molecular family was set to 100, and the lowest scoring edges were removed from molecular families until the molecular family size was below this threshold. The spectra in the network were then searched against GNPS’ spectral libraries. The library spectra were filtered in the same manner as the input data. All matches kept between network spectra and library spectra were required to have a score threshold above 0.60 and at least four matched peaks (MSV000086335, doi:10.25345/C5CJ5B). Clusters detected in blank were removed from the networking that includes spectral data from solvents used in the extraction and chromatographic procedures (dried hexane, chloroform, ethyl acetate, methanol). The molecular networking view and edition were performed in Cytoscape v.3.8.2 [28].

### 2.7. Diaporthe sp. CarGL8 Chromatographic Procedures

**Figure 1:**
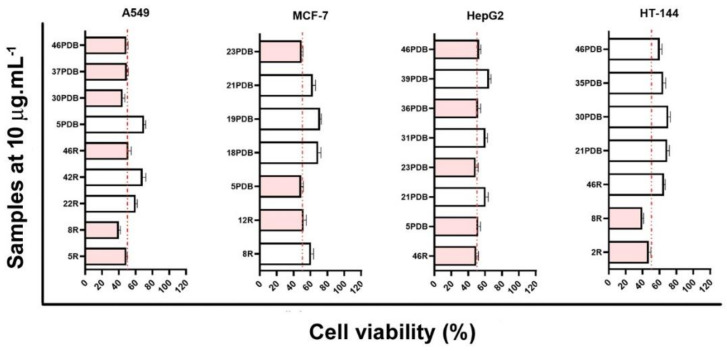
Cell viability of cultured human tumor cell lines with endophytic fungi extracts. Cells were treated for 48 h with crude extracts at 10 µg mL^−1^. Codes next to bars refer to extracts of CarGL strains from R (rice incubation) and PDB (potato dextrose broth incubation). White bars refer to a medium cytotoxic activity, and pink bars refer to high cytotoxic activity.

The extract obtained from *Diaporthe* sp. CarGL8 incubation in rice furnished a brown and amorphous extract in ethyl acetate which presented cytotoxic effects against three tumor cell lines (Figure 1). The crude extract was partitioned with hexane and MeOH:H_2_O (9:1). The hydromethanolic phase (400 mg) was then subjected to a chromatographic column (CC) over SiO_2_ using a gradient in CHCl_3_:MeOH starting at 95:5, increasing the polarity and ending in 100% MeOH furnishing five group fractions (I–V). Group II was selected by the LC-DAD profile for further purification due to the presence of a major compound. Fraction II was subjected to Sephadex LH-20 CC using methanol as the mobile phase furnishing five fractions (II-1 to II-5). The LC-DAD revealed one chromatographic band in group II-1 (90 mg) at 220 nm with a low absorption chromophore UV_max_ 254 nm. The compound II-1 was then analyzed by NMR and HRMS-MS for structural identification.

#### Compound II-1, Cytochalasin H (Cyt-H)

¹H NMR 300 MHz (DMSO-d_6_)—*δ* (ppm): 3.1 (*sl*, 1H, H-3), 1.98 (*m*, 1H, H-4), 2.5 (*m*, 1H, H-5), 3.62 (*d*, 10.0 Hz, 1H, H-7), 2.73 (*d*, 10.0 Hz, 1H, H-8), 1.9 (*dd*, 11.5 Hz, 4.0 Hz, 1H, H-10a), 1.62 (*m*, 1H, H-10b), 0.37 (*d*, 6.6 Hz, 3H, H-11), 4.8 (*s*, 1H, H-12a), 5.0 (*s*, 1H, H-12b), 5.5 (*dd*, 13.7 Hz, 9.4 Hz, 1H, H-13), 5.1 (*ddd*, 9.4 Hz, 4.7 Hz, 1H, H-14), 2.82 (*dd*, 13.7 Hz, 4.7 Hz, 1H, H-15a), 2.56 (*dd*, 13.7 Hz, 9.4 Hz, 1H, H-15b), 1.67 (*m*, 1H, H-16), 1.58 (*m*, 1H, H-17a), 1.40 (*dd*, 13.2 Hz, 2.0 Hz, 1H, H-17b), 5.67 (*dd*, 16.6 Hz, 2.0 Hz, 1H, H-19), 5.37 (*dd*, 16.6 Hz, 2.0 Hz, 1H, H-20), 5.3 (*s*, 1H, H-21), 0.95 (*d*, 6.3 Hz, 1H, H-22), 1.14 (*s*, 3H, H-23), 2.23 (*s*, 3H, H-25), 7.15 (*m*, 2H, H-2′,6′), 7.30 (*m*, 2H, H-3′,5′), 7.22 (*m*, 1H, H-4′), 8.0 (*s*, 1H, NH), 4.5 (*sl*, 1H, 7-OH), 4.38 (*sl*, 1H, 18-OH). ¹^3^C NMR 75 MHz (DMSO-d*_6_*)—*δ* (ppm): 174.0 (C-1), 52.7 (C-3), 47.7 (C-4), 31.6 (C-5), 151.0 (C-6), 70.5 (C-7), 46.1 (C-8), 51.8 (C-9), 42.9 (C-10), 12.9 (C-11), 111.3 (C-12), 128.7 (C-13), 134.5 (C-14), 43.9 (C-15), 27.7 (C-16), 53.8 (C-17), 72.7 (C-18), 125.3 (C-19), 138.0 (C-20), 76.7 (C-21), 26.1 (C-22), 30.9 (C-23), 170.0 (C-24), 20.5 (C-25), 137.2 (C-1′), 129.6 (C-2′,6′), 128.3 (C-3′,5′), 126.5 (C-4′). Spectroscopy data according to the literature [29].

### 2.8. Cell Lines, Cultures Condition, and Viability Assay

Four human cancer cell lines (A549, MCF-7, HepG2, and HT-144) were used in the present study. A549 (non-small cell lung cancer), HepG2 (hepatoma), and MCF-7 (estrogen-positive breast cancer) cell lines were purchased from the Rio de Janeiro Cell Bank. The HT-144 (melanoma) cell line was kindly provided by Dr. Glaucia M. Machado-Santelli from the Institute of Biomedical Sciences (University of São Paulo). Cell cultures were maintained in DMEM (Dulbecco’s modified Eagle’s medium, Sigma, Temecula, CA, USA) supplemented with 10% fetal bovine serum (Vitrocell, Campinas, Brazil). Cells were grown in a humidified atmosphere of 95% air, 5% CO_2_, 37 °C. Cell viability was measured by MTS assay using the CellTiter 96^®^ Aqueous Non-Radiative Cell Proliferation assay (Promega) according to the manufacturer’s instructions. Cells were seeded into a 96-well plate at 1 × 10^4^ cells/well (HepG2, MCF-7, HT144) or 5 × 10^3^ cells/well (A549). After attachment (24 h), the cultures were treated with fungal crude extracts at 10 µg mL^−1^ for 48 h to identify the most cytotoxic extracts. In addition, HepG2, MCF-7, and A549 cells were treated with compound II-1 or cisplatin at different concentrations (0.01–180 µM) for 48 h to determine IC_50_ values. The samples were analyzed in a spectrophotometric plate reader at 490 nm. Relative viability was determined by comparing the amount of formazan produced by metabolically active cells in the control and treated groups. Experiments were conducted in triplicate. Data are presented as the mean ± standard deviation (SD) of three independent experiments. IC_50_ values were determined from nonlinear regression using GraphPad Prism^®^ (GraphPad Software, Inc., San Diego, CA, USA) [30].

## 3. Results

The authors encourage to visit the GNPS platform and re-analyze the MSV000086335 (Supplementary Materials SIX) that contains the HRMS-MS data obtained from this work. Bellow, based on the MN and fragmentation spectra, cytochalasin and octaketides were investigated for the dereplication process to annotate secondary metabolites in the endophytic metabolome.

### 3.1. Cytotoxic Fungi from Casearia arborea Leaves

Forty-seven endophytic fungi were obtained from fresh and healthy leaves of *C. arborea.* The fungi inoculation was conducted in two media cultures: solid using rice (R) and liquid using potato and dextrose broth (PDB), for 28 days, thus furnishing ninety-four extracts in ethyl acetate. The cytotoxic potential for crude extracts was evaluated in front of four human tumor cell lines (A549, MCF-7, HepG2, and HT144). Criteria for the strain selection was cell viability less than 70% as result (Figure 1), comprising to strains CarGL2, 5, 8, 12, 22, 42, 46 from R incubation and strains CarGL5, 18, 19, 21, 23, 30, 31, 35, 36, 37, 39, 46 from PDB incubation. Based on previously cytotoxic strains (CarGL2, 5, 8, 12, 18, 19, 21, 22, 23, 30, 31, 35, 36, 37, 39, 42, 46), both R and PDB extracts of each selected strain were then analyzed in the LC-HRMS-MS system, representing third-four extracts selected for dereplication. The MN was created using the GNPS platform workflow that organized the complex secondary metabolome of twelve *Diaporthe* spp. CarGL2, 5, 8, 12, 18, 19, 30, 31, 35, 36, 37 and CarGL46, three *D. paranensis* (CarGL21, 39 and 42), in addition to *Colletotrichum* sp. (CarGL22) (Supplementary Materials SII). The secondary metabolites from *Phomopsis* sp. CarGL23 [21] were also included in the MN to corroborate octaketide derivatives annotations.

### 3.2. Dereplication Based on Molecular Networking Organization from HRMS-MS Data

GNPS annotations were effective to furnish a broad view of the cytotoxic metabolome of endophytes *Colletotrichum* sp., *Diaporthe* spp., and *Phomopsis* sp. The MN obtained from fungi metabolome were organized according to annotations by GNPS including PKS (polyketide synthase) pathway compounds as lipids, cytosporones (Csn), dothiorelones (Dot), ralfuranone L, as well as alkaloids from PKS and NRPS pathway, cytochalasins (Cyt). One fungal dipeptide was detected and annotated as cyclo(Phe-Leu) (Supplementary Materials SIII–SVIII).

#### 3.2.1. Cytochalasin Dereplication

A molecular family with nodes presenting characteristic fragment patterns of *m*/*z* 398, 416, and 434 suggested loss of water and/or other substituents as methanol or acetic acid that reminds the alkaloid cytochalasin ESI-HRMS-MS spectra [31,32].

The cytochalasin nodes were detected in *Diaporthe* sp. CarGL2, 8, and 37 extracts (Supplementary Materials SIII and SIV). Based on cytotoxic activity against A549, MCF-7, and HepG2 and the annotation of the *m*/*z* 494 cluster as a cytochalasin, *Diaporthe* sp. CarGL8 active extract was selected for chromatographic procedures. The compound II-A (Cyt-H) was obtained from SiO_2_ CC and identified by spectroscopy data according to the literature [29] and ESI-(+)-HRMS-MS. Moreover, a fragment map for II-A (Cyt-H, *m*/*z* 494) was built to annotate cytochalasin derivatives according to the fragmentation mechanisms in the literature [31,32].

**Figure 2:**
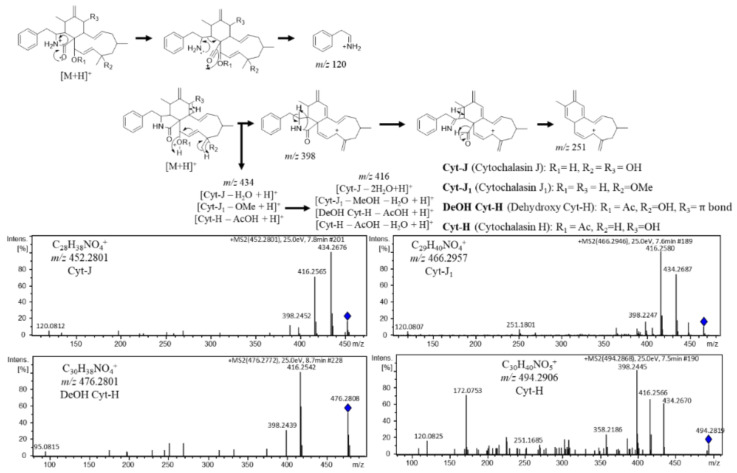
Cytochalasin (Cyt) annotations based on ESI-(+)-HRMS-MS fragmentation mechanisms. The blue diamond inside MS² spectrum represents the precursor ion.

The dereplication process allowed the annotation of Cytochalasin J (Cyt-J, *m*/*z* 452.279), Cytochalasin J_1_ (Cyt-J_1_, *m*/*z* 466.295), and Dehydroxy Cytochalasin H (DeOH Cyt-H, *m*/*z* 476.279). Furthermore, the *m*/*z* 120 ion is also a common fragment according to the loss of biosynthetic precursor phenylalanine moiety. In addition, the node *m*/*z* 434 is related to a cytochalasin fragment created in the ionization chamber, which corroborates with finding the presence of cytochalasin derivatives in active extracts from strains CarGL2 and 37 (Figure 2).

#### 3.2.2. Octaketide Dereplication

The annotation of curvularin (*m*/*z* 293.139) by GNPS aided the identification of a molecular family containing nodes of octaketide derivatives. The neighbor nodes of curvularin presented characteristic HRMS-MS fragment peaks as *m*/*z* 97, *m*/*z* 125, *m*/*z* 275, and *m*/*z* 293, suggesting dothiorelone derivatives. Annotations were based on the fragmentation maps of dothiorelones A or B (Dot-A/B, *m*/*z* 339), dothiorelones P or Q (Dot-P/Q, *m*/*z* 311), and 15-acetoxy dothiorelones A or B (OAc Dot-A/B, *m*/*z* 381) from past work [21].

**Figure 3:**
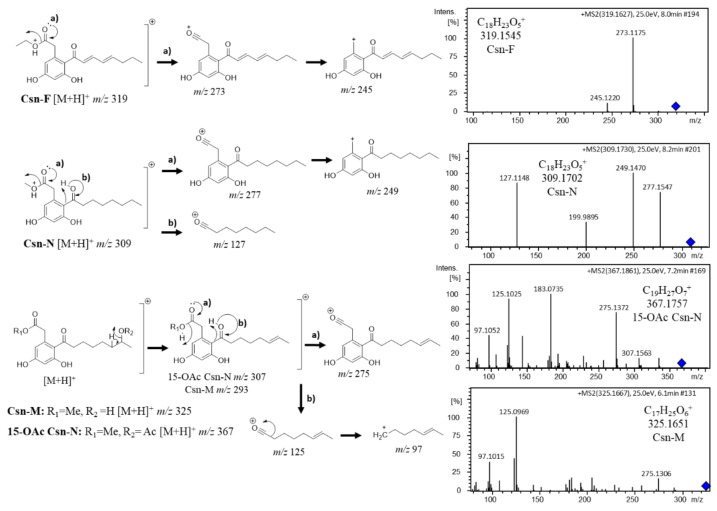
Octaketide (Csn) annotations based on ESI-(+)-HRMS-MS fragmentation mechanisms. The blue diamond in MS² spectrum represents the precursor ion.

Based on ESI-(+)-HRMS-MS fragmentation mechanisms, Cytosporone M (Csn-M, *m/z* 325) and 15-acetoxy cytosporone N (15-OAc Csn-N) annotations were suggested due to a similar lateral chain from Dot-A/B which HRMS-MS presents the *m*/*z* 293 fragment ion by the loss of methanol moiety present in C-15 corresponding to the Csn-M structure or loss of acetic acid furnishing the *m*/*z* 307 fragment ion for 15-OAc for the Csn-N structure. Loss of the charged lateral chain represents the *m*/*z* 125 and *m*/*z* 97 by the loss of CO. Loss of the group linked to carbonyl C-1 furnished the characteristic *m*/*z* 275 ion for both Csn-M and 15-OAc Csn-N (Figure 3). In addition, more nodes presented a dothiorelone base skeleton (*m*/*z* 336, *m*/*z* 353, *m*/*z* 337), as well as curvularin annotation, representing an ESI-(+) fragment according to fragmentation map similarity. Dothiorelones were detected in *Diaporthe* sp. CarGL2, 30 and 37, and *Phomopsis* sp. CarGL23 (Supplementary Materials SV and SVI).

The octaketides Cns-A (*m*/*z* 295.156) and Csn-B (*m*/*z* 323.180), from past work [21], were added to the GNPS MN workflow to corroborate the identity of the molecular family of cytosporone derivatives based on ESI-(+) fragmentation mechanisms [21]. The chemical profile of *Phomopsis* sp. CarGL23 includes the annotations of cytosporone N (Csn-N, *m*/*z* 309.17) according to the common fragment ions from Csn-B fragmentation (*m*/*z* 127, *m*/*z* 249, and *m*/*z* 277), as well as Cytosporone F (Csn-F, *m*/*z* 319.162). The Csn-F annotation was based on the difference of 4 Da in comparison to Csn-B ESI-(+)-HRMS-MS spectra, suggesting a similar structure to Cns-B containing two conjugations in the lateral chain corroborating the ion fragments of *m*/*z* 273 (loss of ethanol) and *m*/*z* 245 (loss of CO from *m*/*z* 273) as observed in Cns-B (Figure 3). At least four cytosporone and five dothiorelone structures annotations based on the HRMS-MS spectra were found in the MN metabolome of *Diaporthe* sp. CarGL2, 8, 30, 37 and *Phomopsis* sp. CarGL23. More clusters neighboring Csn-F suggest cytosporone derivatives (Supplementary Materials SV and SVII).

### 3.3. Cytotoxic Activity of Cytochalasin H

**Figure 4:**
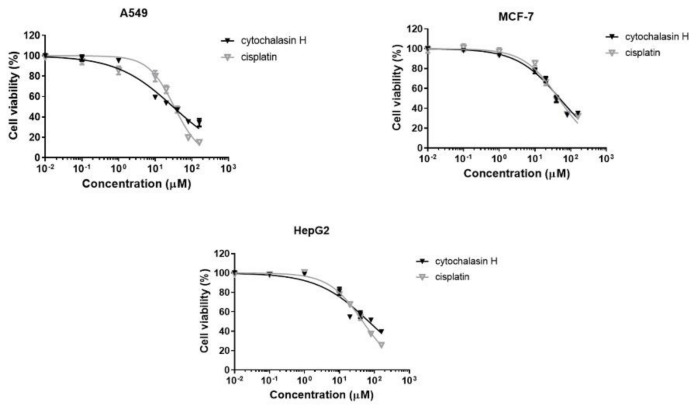
Dose-response curves obtained from viability assay. A549, MCF-7, and HepG2 cells were treated with cytochalasin H or cisplatin at different concentrations for 48 h.

Cell cultures (A549, MCF-7, and HepG2) were treated with compound II-1 at different concentrations for 48 h. The dose-response curves are shown in Figure 4. Cisplatin, a cytotoxic antineoplastic agent, was included in this experimental approach as a positive control. The IC_50_ values found for cytochalasin H and cisplatin are shown in Table 1.

## 4. Discussion

Polyketide derivatives were the major secondary metabolite class found in *Diaporthe* spp. endophytes from *C. arborea*, cited by many researchers as a focus for the search for anticancer natural prototypes [33]. It has been reported that *Phomopsis*, also identified in *C. arborea* mycobiota, has cytotoxic activity against several cancer cell lines including breast cancer (MDA-MB-231 and MCF-7), non-small cell lung cancer (A549), hepatoma (HepG2), neuroblastoma (SHSY5Y), and hematologic cancer (HL-60, K562, and Raji) [34,35,36]. In addition, a study carried out by Cui et al. [37] showed that *Phomopsis* displays inhibitory activity on osteoclastogenesis by suppressing RANKL-induced NF-κB activation.

Lipids, such as phospholipids, phosphocholine, and phytosphingosine derivatives, belong to the PKS pathway and represent an important metabolite class for fungi metabolism. In addition, these compounds are the main components found in fungi biomass [38,39,40]. Polyunsaturated fatty acids (PUFA) also display cytotoxic activity, as reported for PUFA n-3 (Ꞷ-3), a fatty acid known to possess cytotoxic activity against several cancer cell lines, including prostate [41], colorectal [42], and breast cancer cell lines [43,44,45]. *C. arborea* endophytes showed the capability to produce saturated and PUFA lipid derivatives as choline glycerophosphate, LysoPC(16:0), LysoPC(18:2/0:0), and pinolenic acid (Supplementary Materials SVIII).

The octaketides also belong to the PKS pathway presenting a resorcinol lipid skeleton, found in the fungal metabolism of *Phoma*, *Cytospora*, and *Diaporthe*. Natural and synthetic cytosporone B derivatives may be promising prototypes for cancer therapy [46]. These compounds act as an agonist for nuclear orphan receptor TR3, which regulate a diversity of biological processes including proliferation, differentiation, cell survival, and apoptosis [47,48]. TR3 is closely associated with several pathological conditions, such as inflammations, fibrosis, and cancer [49,50,51,52], and it has been considered an important target for drug development [53]. No physiological ligands known for TR3, and Cns-B can regulate its activity. Thus, this compound and others structurally related are of great pharmacological interest [54,55,56,57], and the fungi of *C. arborea* represent a source to explore cytotoxic PKS metabolites for nuclear orphan receptor TR3 assays.

Cytochalasins, alkaloids from hybrid PKS and NRPS pathways, are well known in the metabolism of *Diaporthe* spp. and *Phomopsis* spp. [33]. These compounds were also described in the present work and have been detected in *Diaporthe* sp. CarGL2, 8, and 37 endophyte strains from *C. arborea* leaves. Analyses using LC-HRMS-MS associated with the molecular network have indicated the presence Cyt-H. Furthermore, the isolation and evaluation of the activity of the pure substance were carried out to determine if the substance responsible for the activity observed for the extract was in fact Cyt-H. Thus, after isolation, assays were performed with Cyt-H, and activity was detected against A549 and MCF-7 strains, cell lines not previously investigated, with values comparable to those of the standard drug cisplatin (Table 1). Additionally, cytochalasin derivatives are described as acting in actin filaments causing cytokinesis inhibition. At high concentrations, cytochalasin is strongly cytotoxic by inducing the loss of cell denucleation [58,59]. Recently, three cytotoxic Cyt-H derivatives (4′-hydroxy-deacetyl-18-deoxycytochalasin H, deacetyl-18-deoxycytochalasin H, and 18-deoxycytochalasin H) were described from the endophyte *Trichoderma harzianum*. The cytotoxic assays showed that human cell lines L5178Y and A2780 were most sensitive to 18-deoxycytochalasin H with IC_50_ values of 0.19 μM and 0.42 μM, respectively [60]. From *Eutypella scoparia,* PSU-H267 metabolism (obtained from the leaves of *Hevea brasiliensis*) was identified Scoparasin C, a cytochalasin derivative that was active against Vero cell lines with IC_50_ values of 1.19 µM [61]. Li and colleagues (2018) described that alkaloids and nitrogen-containing natural products are mainly found in endophytic fungi and reported strong cytotoxic activities against several tumor cell lines [7]. Alkaloid, peptide, and nitrogen compounds from fungi have been described with a broad range of biological properties including anticancer activity [6,7,62]. The purification of cytotoxic compound II-A (Cyt-H) targeted and guided by HRMS-MS provided a good integration with bio-guided assays.

In conclusion, the IC_50_ values of Cyt-H in tumor cell lines were evaluated and led to good expectations of this substance as an antitumor drug prototype. Thus, the work showed that molecular networking is useful to understand the chemical profile of complex matrices enabling the isolation of target compounds, in our case Cyt-H, as well as minimizing cost and time spent in purification processes. In addition, with the complement of tests using a pure compound, it was possible to determine the real antitumor potential of the *Diaporthe* fungus extract.

## Figures and Tables

**Table 1:** IC_50_ values (µM) determined after 48 h treatment.

	A549	MCF-7	HepG2
Cytochalasin H	31.0 ± 3.2	49.2 ± 4.2	71.2 ± 11.0
Cisplatin	33.2 ± 1.2	45.8 ± 0.8	46.6 ± 0.9

## Data Availability

All Mass Spectrometry data used in this work is available at MassIVE repository MSV000086335 according the DOI code 10.25345/C5CJ5B. The repository is accessible online (https://massive.ucsd.edu/ProteoSAFe/static/massive.jsp, accessed on 15 September 2022). For re-analysis the GNPS platform is also accessible (https://gnps.ucsd.edu/ProteoSAFe/static/gnps-splash.jsp, accessed on 15 September 2022).

## Supplementary Materials

The following supplementary information material can be downloaded at: https://www.mdpi.com/article/10.3390/metabo12100903/s1, Supplementary Materials SI–SIX.
