# Supplementary material for: Dereplication of Cytochalasans and Octaketides in Cytotoxic Extracts of Endophytic Fungi from Casearia arborea (Salicaceae)
Source: Metabolites. 2022 Sep 26;12(10):903. doi: 10.3390/metabo12100903 (PMC9611219; doi:10.3390/metabo12100903)
Supplement: Supplementary file 1 [file metabolites-12-00903-s001.zip › metabolites-1925523-supplementary.pdf]

SUPPLEMENTARY INFORMATION MATERIAL

Dereplication of Cytochalasans and Octaketides in Cytotoxic Extracts of Endophytic Fungi from *Casearia arborea* (Salicaceae)

Augusto L. Santos [1], Marisa Ionta [2], Renato O. Horvath [2], Marisi G. Soares [3], Daniele O. Silva [3], Eunizinis S. Kawafune [4], Marcelo J. P. Ferreira [4,*] and Patricia Sartorelli [1,*]

Table of contents

Suppl. Material	*Title*	*Page*
Suppl. I	Phylogenetic tree for *Diaporthe* sp.	1
Suppl. II	Molecular Networking	2
Suppl. III	Cytochalasan derivative annotations	3
Suppl. IV	Molecular family of cytochalasan.	4
Suppl. V	Octaketide derivatives annotations from *C. arborea* endophytes.	5
Suppl. VI	Molecular family of dothiorelone.	6
Suppl. VII	Molecular family of cytosporone.	7
Suppl. VIII	Other compounds annotations.	8
Suppl. IX	GNPS Data Analysis Guide.	9

Suppl. SI: Phylogenetic tree generated by the analysis of maximum parsimony and bayesian sequences of the ITS region of the rDNA of Diaporthaceae species with the se-lected strains (highlighted in gold). Support values are maximum parsimony and bayes-ian analysis, respectively. To the left of the name of each species is the access code to Gen-bank.

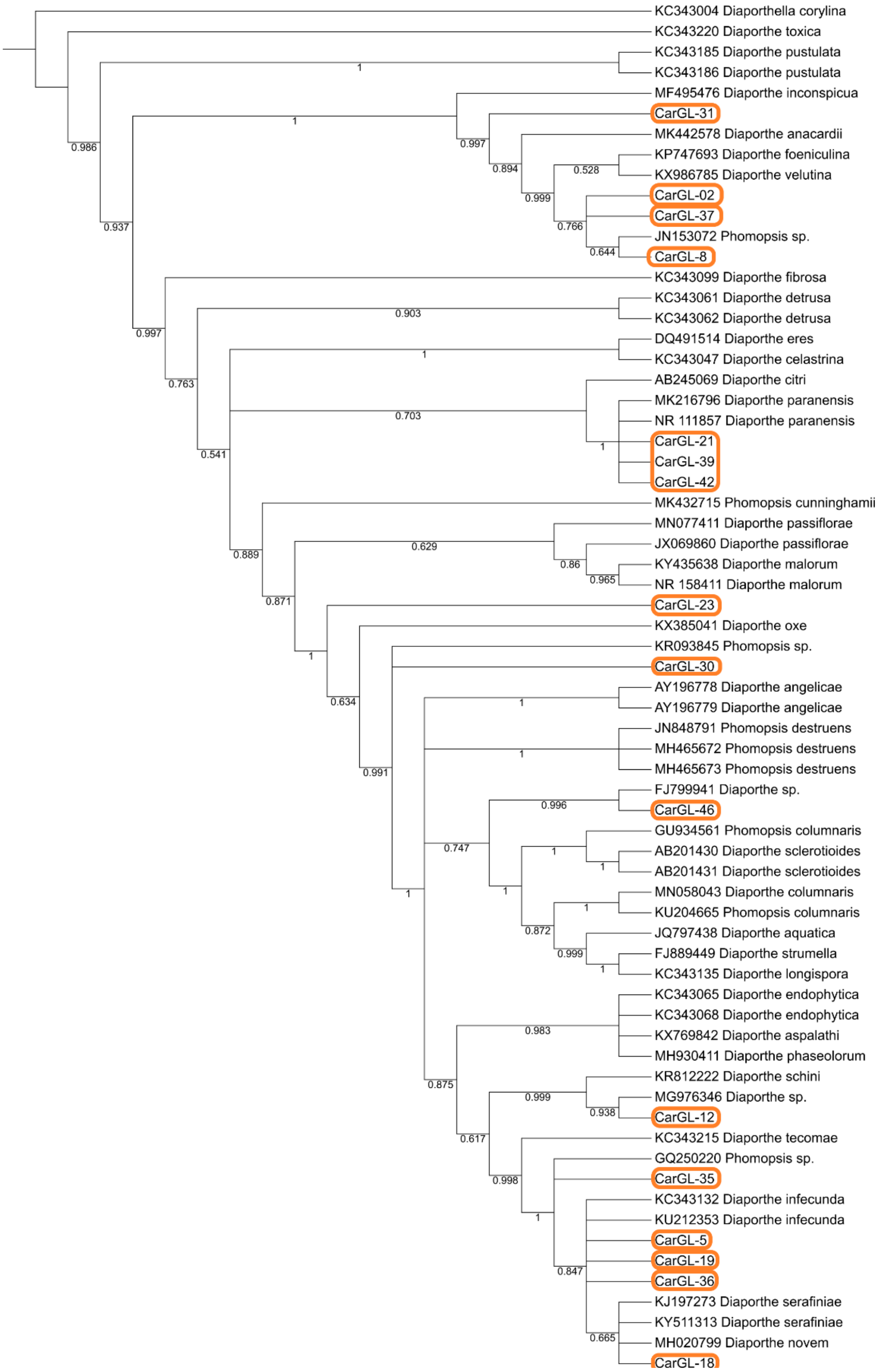

Suppl. SII: Metabolome of endophytes organized in molecular networking format based on extracts from the endophytic fungi incubation in R and PDB. The metabolic clas-ses are emphasized bellow the respective molecular family in the left, as the unknown metabolites cluster spectra are represented in the right. Colors represents groups G1 to G4 as follows: *Phomopsis* sp. CarGL23 (G1 pink), *Colletotrichum* sp. (G2 blue), *Diaporthe* spp. (G3 green) and *Diaporthe paranensis* (G4 orange). Spectra detected in medium culture and blank were removed from MN.

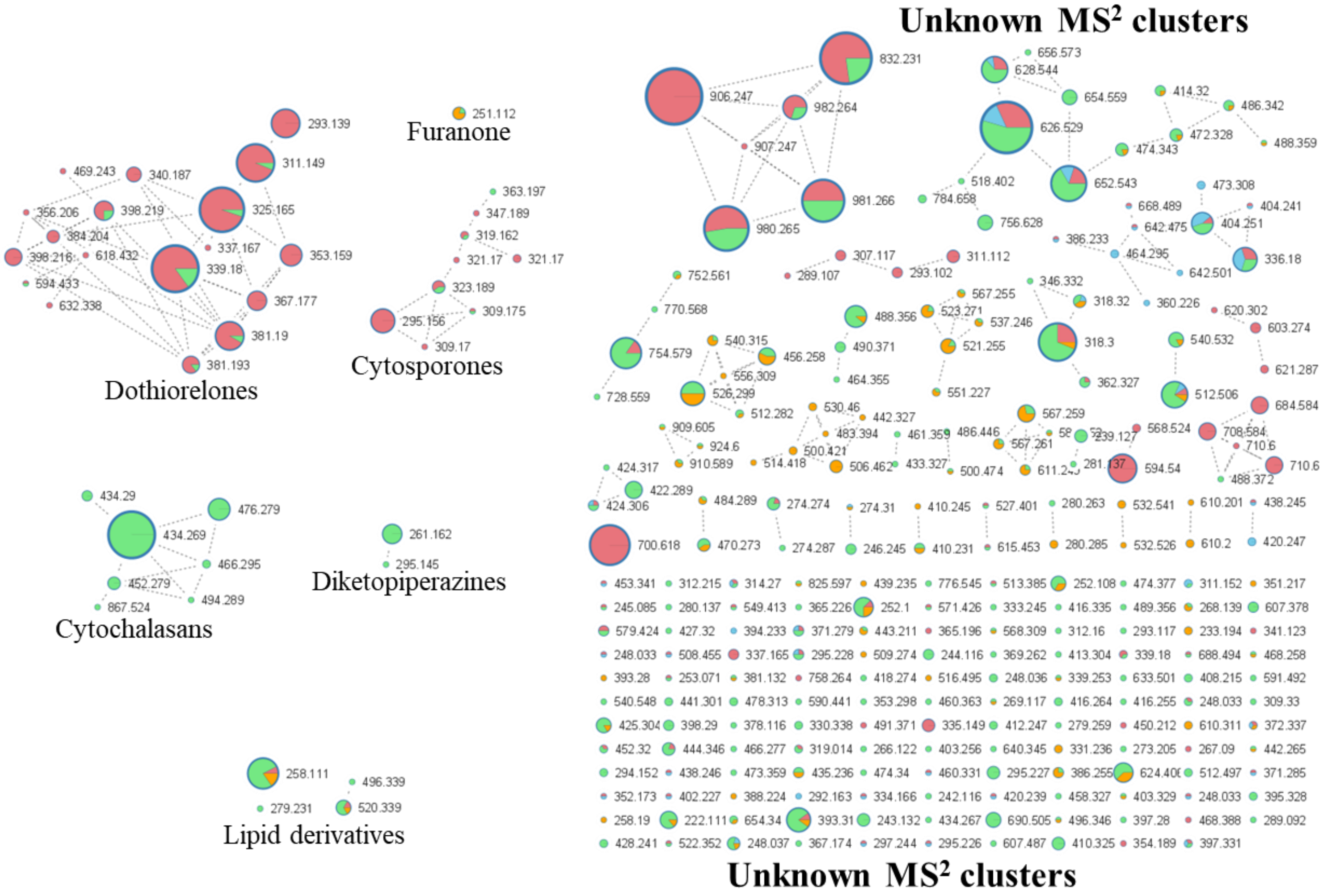

Suppl. SIII: Cytochalasan derivatives annotations from *C. arborea* endophytes.

Cluster Index	Strain	Name	QTof	ESI-(+)
1115	2R, 8R	Cytochalasin J (Cyt-J)	434, 416, 398, 251, 120	452.279
1116	37R		434, 416, 398, 251, 120	452.305
1187	2R, 8R	18-Methoxy Cytochalasin J (Cyt-J1)	434, 416, 398, 251, 120	466.295
1224	37R	18-Deoxy Cytochalasin H (18-Deoxy Cyt-H)	416, 398, 251, 120	476.279
1227			416, 398, 269, 242, 120	476.298
1281	8R	Cytochalasin H (Cyt-H)	476, 434, 416, 398, 392	494.289
2114	2R, 8R	2 x 433 [2M+H]$^+$	434, 416, 398	867.524

Suppl. SIV: Molecular family of cytochalasan.

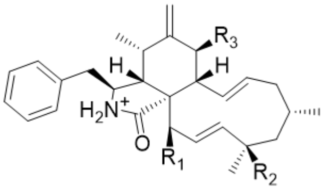

Cyt-J (R_1 = OH, R_2 = OH, R_3 = OH) *m/z* 452.280
Cyt-J_1 (R_1 = OMe, R_2 = OH, R_3 = OH) *m/z* 466.296
18-Deoxy Cyt-H_1 (R1 = OAc, R_2 = H, R_3 = O) *m/z* 476.280
Cyt-H (R_1 = OAc, R_2 = OH, R_3 = OH) *m/z* 494.291

Cyt-J: *Diaporthe* 2R, 8R, 37R
Cyt-J_1: *Diaporthe* 2R, 8R
18-Deoxy Cyt-H: *Diaporthe* 2R, 8R, 37R
Cyt-H: *Diaporthe* 8R

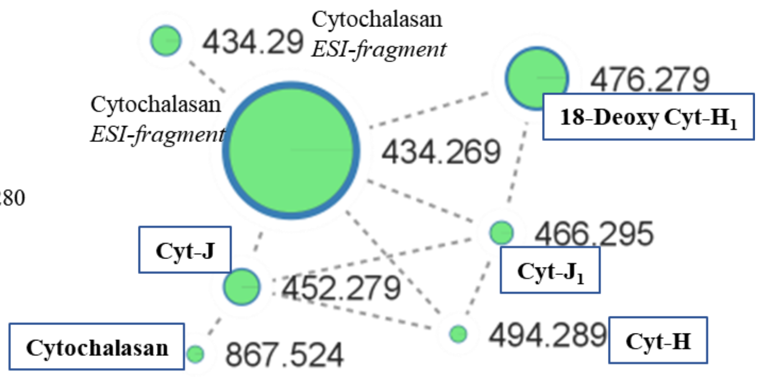

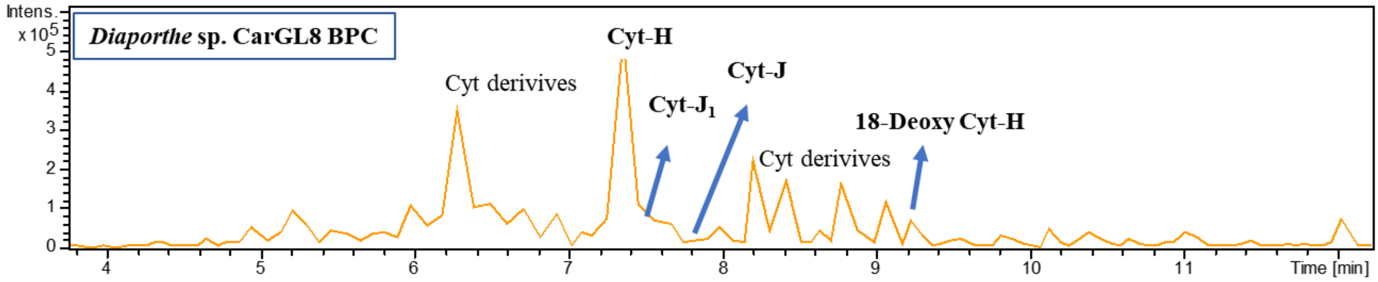

Suppl SV: Octaketide derivatives annotations from *C. arborea* endophytes.

Cluster Index	Strain	Name	Cosine threshould	*m/z* error (ppm)	[M+H]$^+$
295	23R	Curvularin (Dot ESI fragment)	0.80	3	293.139

Dothiorelone derivatives manual annotations

Cluster Index	Strain	Name	QTof	ESI-(+)
428	23R, 8R	Dothiorelone P or Q (Dot-P/Q)	275, 125, 97	311.149
514	23R, 8R	Cytosporone M (Csn-M)	275, 125, 97	325.165
580	23R	**Dothiorelone derivative**	275, 257, 125(123), 95(97)	337.167
601	2R, 8R, 23R	Dothiorelone A or B (Dot-A/B)	275, 125, 97	339.180
603	30R, 37R		293, 257, 249,125, 97	339.201
652	23R	**Dothiorelone derivative**	293, 275, 257, 125, 97	353.159
723	23R	15-Acetoxy Cytosporone N (15-OAc Csn-N)*	307, 275, 125, 97	367.177

780, 781	23R, 30R	15-Acetoxy Dothiorelone A (15-OAc Dot-A)	275, 125, 97	381.193
Cytosporone derivatives manual annotations				
321	23R	Cytosporone A (Csn-A)	277, 249, 181, 127, 123, 109	295.156
396	23R	Cytosporone N (Csn-N)	277, 249, 127, 109	309.170
501	23R	OAc-Dot A/B ESI fragment	275, 247, 125	321.170
492	23R, 30R	Cytosporone F (Csn-F)	273, 245, 123, 107	319.162
508	23R, 30R	Cytosporone B (Csn-B)	277, 249, 127, 109	323.189

*Reference: Beekman, A.M. and Barrow, R.A. (2015) Syntheses of cytosporones A, C, J, K, and N, metabolites from medicinal fungi. *Australian Journal of Chemistry* **68,** 1583-1592.

Suppl SVI: Molecular family of dothiorelone.

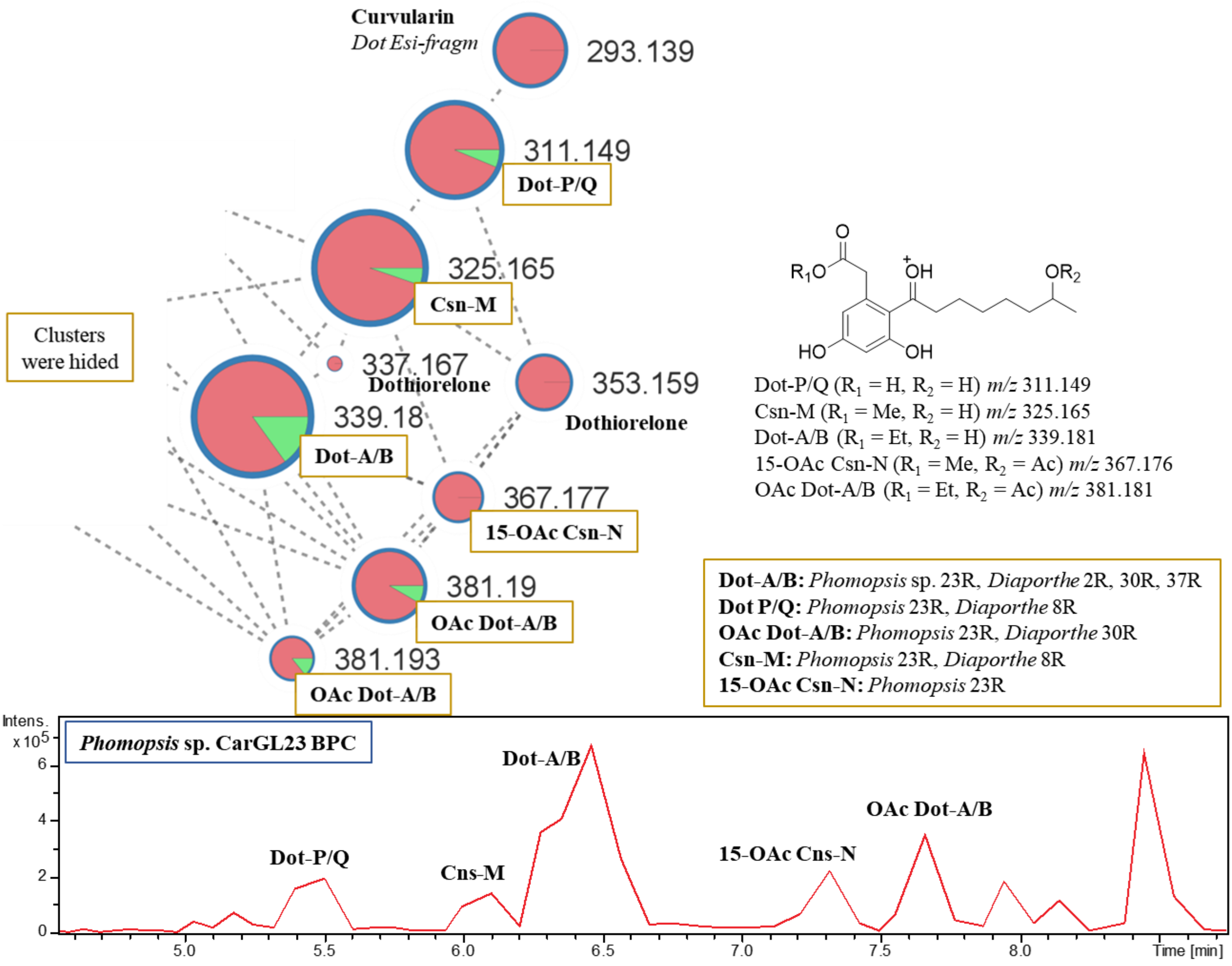

Suppl. SVII: Molecular family of cytosporone.

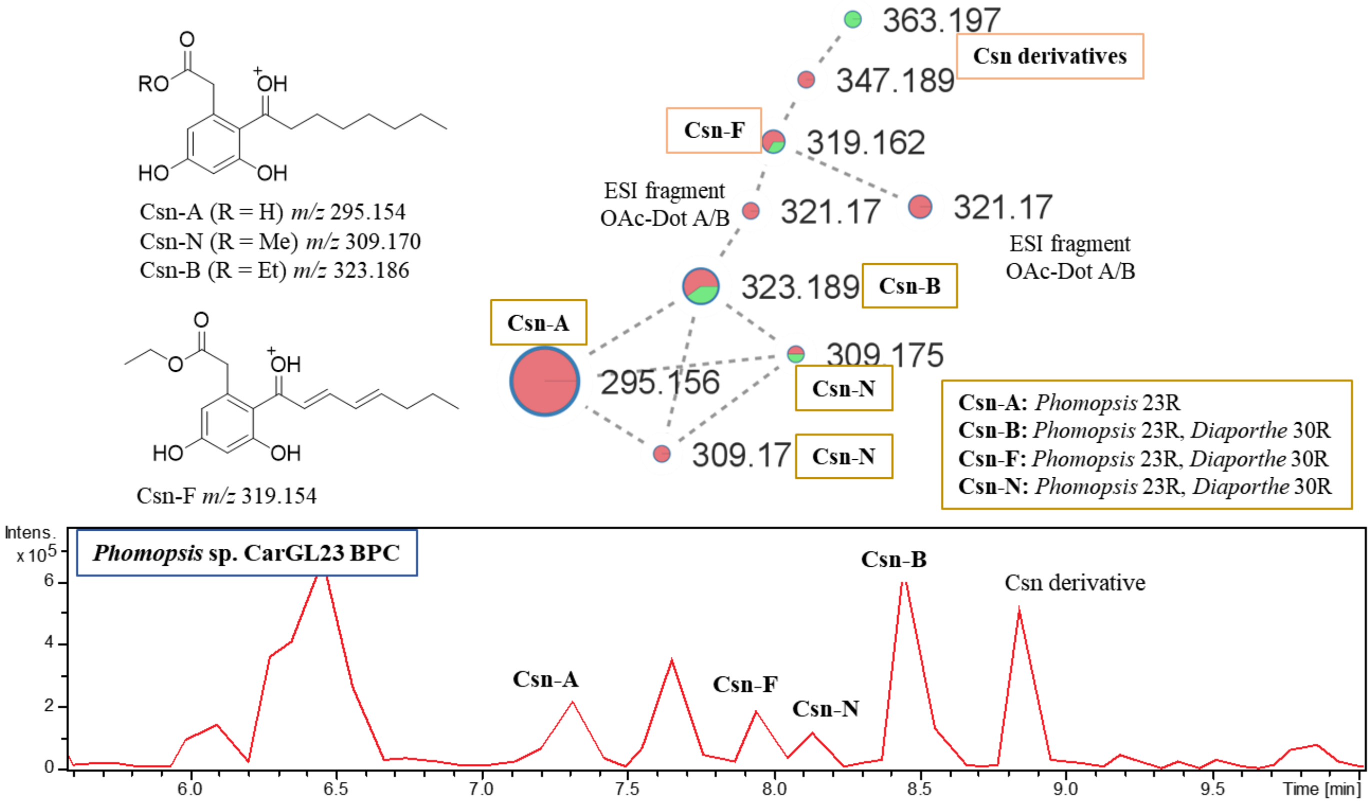

Suppl. SVIII: Other compounds annotations from *C. arborea* endophytes.

Cluster Index	Strain	Name	Cosine threshould	*m/z* error (ppm)	$[M+H]^+$
111	21R<br>2PD, 21PD, 39PD, 42PD	Ralfuranone L	0.78	19	251.112
153	2R, 5R, 18R, 19R, 35R, 36R, 37R, 46R	cyclo(Phe-Leu)	0.72	11	261.162
137	2R, 5R, 19R, 21R, 23R, 30R, 35R, 36R, 39R, 46R<br>5PD, 18PD, 36PD	Choline glycerophosphate	0.91	3	258.111
1289	2R, 46R	LysoPC(16:0)	0.90	0	496.339
1378	2R, 21R, 23R, 46R<br>18PD, 19PD	LysoPC(18:2/0:0)	0.86	3	520.339
229	19R, 35R	Pinolenic acid	0.69	3	279.231

Suppl. SIX: GNPS Data Analysis Guide

"A" files: rice incubation; "B" or "BD": potato dextrose broth incubation.

MassIVE Dataset: MSV000086335.
Casearia arborea's endophytic fungi active extracts [doi:10.25345/C5CJ5B].

Data Analysis

Basic options

- Mass tolerance for parent ions and fragment ions: 0.02

- G1: *Phomopsis* sp. CarGL23
- G2: *Colletotrichum* sp. CarGL22
- G3: *Diaporthe* spp.
- G4: *Diaporthe paranensis*
- G5: Rice, PD broth
- G6: blank (concentrated solvents from chromatographic procedures, diluted in methanol:acetonitrile 1:1)

Advanced Molecular Networking options

- Cosine: 0.7
- Minimum matched peaks: 3
- Cluster size: at least 2 spectra
- Network TopK: 10
- Max. connected component size: 100
- Google Sheets Metadata URL:

Advanced Library Search options

- Score threshold: 0.65
- Matched peaks: 4
- Search analogs: Don't search
- Max. analog search mass diff: 100.0

Advanced Filter options

- Filter below Std Dev: 0
- Min. peak intensity: 0
- Filter precursor window: Filter
- Filter library: filter library
- Filter peaks in a 50 Da window: Filter
- Filter spectra from G6 as blank to remove them from networking: Filter
